# Improved indel detection in DNA and RNA via realignment with ABRA2

**DOI:** 10.1093/bioinformatics/btz033

**Published:** 2019-01-15

**Authors:** Lisle E Mose, Charles M Perou, Joel S Parker

**Affiliations:** 1 Lineberger Comprehensive Cancer Center, University of North Carolina at Chapel Hill, Chapel Hill, NC, USA; 2 Department of Genetics, University of North Carolina at Chapel Hill, Chapel Hill, NC, USA

## Abstract

**Motivation:**

Genomic variant detection from next-generation sequencing has become established as an extremely important component of research and clinical diagnoses in both cancer and Mendelian disorders. Insertions and deletions (indels) are a common source of variation and can frequently impact functionality, thus making their detection vitally important. While substantial effort has gone into detecting indels from DNA, there is still opportunity for improvement. Further, detection of indels from RNA-Seq data has largely been an afterthought and offers another critical area for variant detection.

**Results:**

We present here ABRA2, a redesign of the original ABRA implementation that offers support for realignment of both RNA and DNA short reads. The process results in improved accuracy and scalability including support for human whole genomes. Results demonstrate substantial improvement in indel detection for a variety of data types, including those that were not previously supported by ABRA. Further, ABRA2 results in broad improvements to variant calling accuracy across a wide range of post-processing workflows including whole genomes, targeted exomes and transcriptome sequencing.

**Availability and implementation:**

ABRA2 is implemented in a combination of Java and C/C++ and is freely available to all from: https://github.com/mozack/abra2.

**Supplementary information:**

Supplementary data are available at *Bioinformatics* online.

## 1 Introduction

Next generation sequencing (NGS) has become a widely used tool for a variety of applications. Variant calling has been an area of great interest in DNA for some time and is of increasing interest in RNA. One of the first steps in a NGS variant calling pipeline is to align sequenced reads to a reference genome. Widely used DNA aligners such as bwa-mem and bowtie2 (Langmead and Salzberg, 2012; Li, 2013) are capable of quickly aligning large numbers of reads and support gapped alignment, thus allowing identification of indels. For the sake of speed, these methods do not align each read exhaustively and may in some cases fail to reveal indels, particularly at increased indel lengths. Historically, variant callers have relied upon accurately mapped reads to identify Single Nucleotide Variants (SNVs) and indels. Instances where the reads are not accurately mapped can confound variant detection.

In recent years, a number of methods have been developed that offer improved detection of indels in DNA. In some cases, variants can be successfully identified as long as the reads containing the variant are mapped to roughly the correct location. The original ABRA implementation (Mose *et al.*, 2014) used a localized assembly process to adjust read alignments, thus revealing indels in the alignments and improving variant detection in a variety of callers such as Freebayes (Garrison and Marth, 2012) for calling inherited variants, and Strelka for somatic calling (Saunders *et al.*, 2012). Recently developed callers used for inherited variant detection such as Platypus, GATK-HaplotypeCaller (GATK-HC), Strelka2 and Scalpel (DePristo *et al.*, 2011; Kim *et al.*, 2018; Narzisi *et al.*, 2014; Rimmer *et al.*, 2014) all make use of localized assembly allowing for detection of indels that may or may not be included in the original read alignments. Similarly, the recently developed Lancet and Mutect2 (Cibulskis *et al.*, 2013; Narzisi *et al.*, 2018) make use of localized assembly for somatic variant calling.

RNA-Seq has proven to be extremely important as a diagnostic tool allowing for analysis of gene and isoform expression, gene fusions, transcript splicing, expressed variation and RNA editing. The presence of splice junctions in RNA necessitated the development of splice aware aligners. Several RNA-Seq aligners have been developed that are capable of mapping reads that span splice junctions (Dobin *et al.*, 2013; Kim *et al.*, 2013, 2015; Wang *et al.*, 2010; Wu *et al.*, 2016). However, the presence of non-trivial variation can cause RNA-Seq reads to not entirely align. For example, Sun *et al.* have demonstrated that standard RNA-Seq pipelines have difficulty identifying indels of length greater than 2 bases (Sun, 2016). Frequently, variant calling pipelines that were originally developed for DNA are modified to handle the syntax for RNA-Seq alignments, but are not optimized for RNA-Seq. For example, the widely used GATK (DePristo *et al.*, 2011) requires RNA-Seq read alignments to be broken into multiple alignments with the splice junctions removed, producing DNA-like reads that can then be processed using tools originally developed for DNA variant calling. Additionally, the recently developed Transindel (Yang *et al.*, 2018) relies upon bwa-mem—a DNA aligner—for the initial alignment of reads.

ABRA2 is an update to the original ABRA implementation that provides splice-aware re-alignment of RNA-Seq data as well as substantially enhanced computational performance. The improved alignments produced by ABRA2 enable more accurate variant calling of expressed variants in general, and for indels in particular. Furthermore, ABRA2 improves upon the original ABRA’s accuracy on DNA, offers faster run times, and enables enhanced scalability capable of handling human whole genomes.

## 2 Algorithm

### 2.1 Original Abra implementation

Briefly, the original ABRA implementation processes input BAM files on a localized per region basis. Reads of each region of interest are extracted and assembled using a deBruijn graph. Assembled contigs are added to a global pool of contigs. Once assembly is complete across all regions, all contigs are then aligned to the reference genome using bwa-mem. Chimeric alignments of a given contig are combined when the alignments clearly indicate the presence of an indel that can be simply represented as a single variant. Once all contigs have been aligned, bwa-mem is again used to map all reads to all contigs. When a read maps more closely to a contig than the reference, it is updated to match the contig alignment in the context of the reference.

While this method has proven to be effective in many cases of targeted DNA sequencing such as gene panels and whole exomes, there are several clear shortcomings. When the total number of contigs grows large, aligning all reads to all contigs can become prohibitively slow and in some cases cause bwa-mem to not run to completion. This causes problems in scalability as well as computational difficulties for noisy samples and samples that diverge substantially from the reference genome as is the case for many mouse strains. Due to the scalability problems, large samples such as human whole genomes typically fail to run to completion. Post processing of chimeric bwa-mem alignments may work well in the presence of a single, simple isolated indel, but may not properly capture more complex or noisy events consisting of multiple variants or nearby technical artifacts. Localized assembly has become widely used in variant calling, but is computationally expensive and may not always be necessary to identify pertinent variants resulting in unnecessarily long compute times. Finally, neither the original ABRA nor bwa-mem account for splicing, thus rendering the tools suboptimal for RNA-Seq data. We have developed ABRA2 to ameliorate these issues.

### 2.2 Realignment

#### 2.2.1 Overview

**Fig. 1.**
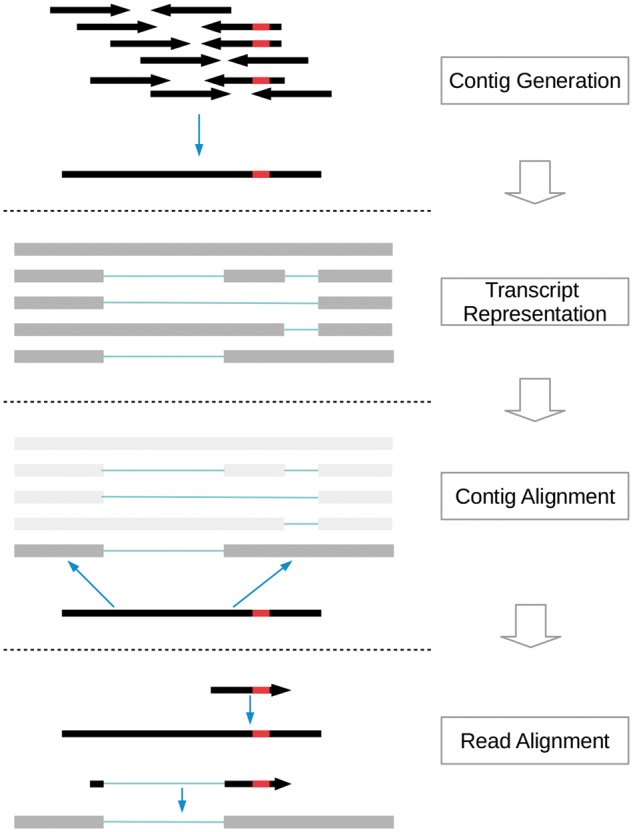
Overview of ABRA2 workflow. Reads overlapping a genomic window of 400 bp are extracted. Contigs are generated using a variety of mechanisms including localized assembly, identification of substantially soft clipped reads, placement of observed indels in localized reference representations and input known indels. Reference representations of putative transcripts are generated using annotated splice junctions as well as unannotated splice junctions observed in the original read alignments. Contigs are exhaustively mapped to each transcript/reference representation using semi-global alignment and the single best alignment is identified. Reads are then aligned back to the contigs using a simple seed and extend approach. If a read unambiguously aligns more closely to a contig than the reference, then the read alignment is updated based upon the contig’s alignment to the reference

ABRA2 operates on a per-region basis analyzing windows of size 400 bp with each window overlapping by 200 bp. Either the entire genome is traversed, or regions of interest can be specified via a bed file. Contigs are generated for each region of interest and aligned back to the reference. Reads are then mapped to the generated contigs and updated in cases where an improved alignment is discovered (Fig. 1).

#### 2.2.2 Contig generation

Candidate contigs representing variation from the reference for a given region are generated in a variety of ways.

1. Indels and/or splice junctions observed in the original read alignments inserted into a localized reference representation.
2. Sequence extracted from reads containing substantial high quality soft clipping (15 or more bases). Reads aligning to the same locus may optionally be merged to form a consensus representation which may be of benefit in cases of amplicon sequencing.
3. Contig generation via localized assembly (Pevzner *et al.*, 2001) of reads mapped to the region of interest along with unmapped reads anchored by their pairs. The assembly is executed when the fraction of assembly triggering reads exceeds a configurable fraction of total reads in the region. Assembly triggers include read pairs with one read unmapped, insertions longer than 15% of the read length in the original alignments, or the presence of high quality soft clipped bases longer than 25% of the read length. In cases where multiple samples are present (including matched tumor/normal pairs), they are assembled jointly.
4. Known or suspected indels passed in as an input VCF file inserted into a localized reference representation.

#### 2.2.3 Transcript/Reference representations

All splice junctions with a start or end point within the region of interest (padded by 2 read lengths on either end) are identified to create a local set of splice junctions J. These may include annotated splice junctions as well as splice junctions identified in the input read alignments. Additionally, splice junctions within a read length of the start or end point of any member of J are added to J. This step is repeated a second time, which ultimately has the net effect of allowing reads that partially overlap the region of interest to span 2 splice junctions outside the region of interest and still map properly. Once the junctions in J are determined, all possible combinations of the elements of J are identified recursively. Combinations that do not contain overlapping splice junctions are considered to be a valid potential transcript. While identifying all possible combinations of splice junctions can result in substantially more putative transcripts than simply utilizing annotated transcripts, this is necessary to enable accurate alignment of reads in the presence of alternative transcripts that may not be annotated. Reference representations of each transcript are generated based upon the input reference genome utilizing the loci of the splice junctions corresponding to the putative transcript.

#### 2.2.4 Contig alignment

Contigs generated from the previous step are then aligned to the localized regional representations of the reference using semi-global alignment with affine gap penalties (Brudno *et al.*, 2003; Gotoh, 1982; Smith and Waterman, 1981). The optimal end to end alignment of each contig is identified within the local reference representation. When multiple putative transcripts are present, each contig is aligned to each transcript representation and the single highest scoring alignment is selected. The final alignment is modified to include junctions present in the best localized reference representation.

#### 2.2.5 Read alignment

Reads that do not perfectly match the reference are mapped to each aligned contig using a simple ungapped seed and extend alignment. The regional reference representation is hashed into 10-mers which serve as the seeds. A read is compared to all potential reference positions with a matching seed with the smallest number of mismatches indicating the optimal alignment. Unlike the original ABRA implementation, reads are only remapped to contigs arising from nearby regions. If a read unambiguously maps to a contig more closely than the reference, the read is updated using the contig’s alignment in the context of the reference.

### 2.3 Variant calling

Included with ABRA2 is a simple somatic indel caller named Cadabra that is capable of calling somatic indels on ABRA2 realigned BAM files. Reads that map unambiguously to an indel arising from a contig are used to gather variant counts. These reads are identified using SAM tags inserted during realignment. Fisher’s Exact Test is used to evaluate somatic variant status in a fashion similar to Varscan2 (Koboldt *et al.*, 2012). In the presence of repeats, only reads that span the full repeat are evaluated (Gymrek *et al.*, 2012). Additional simple filters are used to filter variants including a read orientation bias filter similar to that implemented in GATK, a low positional read complexity filter based upon the difference of the max and min starting position of a variant in the supporting reads and a filter for variant loci where a majority of spanning reads have a mapping quality of zero. Optional variant quality penalties are applied at loci with short tandem repeats and homopolymer runs to accommodate increased errors that may be caused by slippage during Polymerase Chain Reaction (PCR) amplification.

## 3 Results

### 3.1 Evaluation of somatic DNA variant calling

**Fig. 2.**
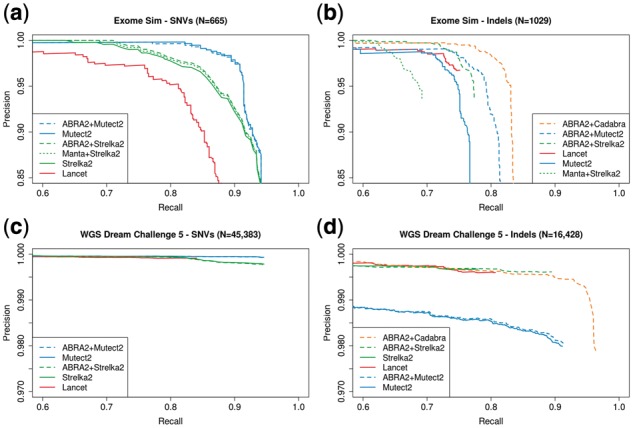
Evaluation of somatic variant calling in DNA. (**a**) Precision and recall of somatic SNV detection on a challenging simulated exome dataset containing insertions and deletions ranging in length from 1 to 100 bp and variant allele frequency ranging from 10 to 50%. Strelka2, Strelka2/Manta, Mutect2 and Lancet are evaluated with Strelka2 and Mutect2 also applied to ABRA2 realignments. (**b**) Precision and recall of somatic indel detection on the exome dataset. In addition to the SNV callers, Cadabra is applied to the ABRA2 realignments. Pipelines including ABRA2 produce the best overall results. (**c**) Somatic SNV calling on the ICGC Dream Somatic Mutation Calling Challenge 5 dataset. Strelka2, Mutect2 and Lancet are evaluated with Strelka2 and Mutect2 also applied to ABRA2 realignments. (**d**) Somatic indel calling on the ICGC Dream Somatic Mutation Calling Challenge 5 dataset. In addition to the SNV callers, Cadabra is applied to the ABRA2 realignments. ABRA2 combined with Cadabra or Strelka2 produces the best overall results

To assess the performance of ABRA2 on somatic DNA calling we begin with a simulated matched tumor/normal exome dataset. To create a challenging dataset, 665 SNVs and 1092 indels were spiked in at variant allele frequencies of 10, 25 and 50%, and indel lengths ranging from 1 to 100 bp with a median length of 31 bp. Variants were called using Mutect2 and Strelka2, both with and without ABRA2 alignments. Lancet variants were called without ABRA2. ABRA2 has a small effect on SNV detection (Fig. 2a) and enables a substantial improvement in indel detection (Fig. 2d). The Cadabra+ABRA2 combination produces superior accuracy to all other methods. Mutect2 and Strelka2 show a substantial improvement in accuracy when using ABRA2 realignments compared to Mutect2 alone and Strelka2 run in conjunction with Manta (Chen *et al.*, 2016). Notably, the three ABRA2 cases show improved indel detection accuracy compared to all other methods, with Lancet as the next best performing method. Compared to the original ABRA implementation, ABRA2 provides increased sensitivity for indel detection on this dataset (Supplementary Fig. S1). The ABRA2 runtime on this dataset was 39 min using 8 cores compared to 1 h 45 min for the original ABRA.

We also assessed the performance of ABRA2 on this dataset both with and without assembly. Notably, high accuracy is achievable without utilizing localized assembly although assembly does offer a boost in recall for longer insertions. The version of ABRA2 run with localized assembly disabled detected 17 fewer insertions with a median length of 60 nucleotides. All other variants were detectable without the use of assembly (Supplementary Fig. S2). The runtime for ABRA2 with localized assembly disabled was 32 min. By contrast, when assembly is forced to execute across all target regions, the ABRA2 runtime was 610 min. This assemble all regions approach detected no additional variants detected compared to ABRA2 run with selective assembly. While localized assembly can be beneficial for variant detection, from a computational performance standpoint it can be helpful to perform this step selectively.

We assessed the impact of utilizing known indels on this dataset by running ABRA2 with the truth set of indels passed as candidate loci to the realigner. As expected, we observed improved accuracy when utilizing known indel information and most calls revealed by using known indels as input are longer insertions (median 49 bases) (Supplementary Fig. S3). A majority of these calls are local repeats (26 out of 31). Local repeats are likely to map to the region of interest, but can confound assembly due to cycles in the graph. An additional 52 long insertions (median length 61.5) of the correct length and position were called after realignment with known indels, but not at nucleotide resolution. Over 90% of these calls were insertions of sequence arising from a distant location of the genome (i.e. mobile insertions). By comparison, there were eight true positive calls not at nucleotide resolution in the ABRA2 de novo dataset all of which are mobile insertions. In contrast with local repeats, reads that span a mobile insertion with a length greater than half of the read length are likely to align elsewhere in the genome resulting in no reads spanning the full insertion in the region of interest, thus causing difficulty in variant identification for localized processing. All non-nucleotide resolution calls were filtered from the result set in the precision/recall plots.

We also evaluated ABRA2 on the somatic whole genome ICGC-TCGA-DREAM Somatic Mutation Calling Challenge #5 dataset (Ewing *et al.*, 2015). As with the exome dataset, ABRA2 has minimal impact on SNV detection (Fig. 2c). Indel sizes on this dataset are generally smaller with a median length of 7 bp and as a result recall is higher among all methods (Fig. 2d). Cadabra+ABRA2 and Strelka2+ABRA2 are the top performers in this dataset with ABRA2 enabling a marked improvement in recall.

### 3.2 Evaluation of RNA-Seq variant calling

**Fig. 3.**
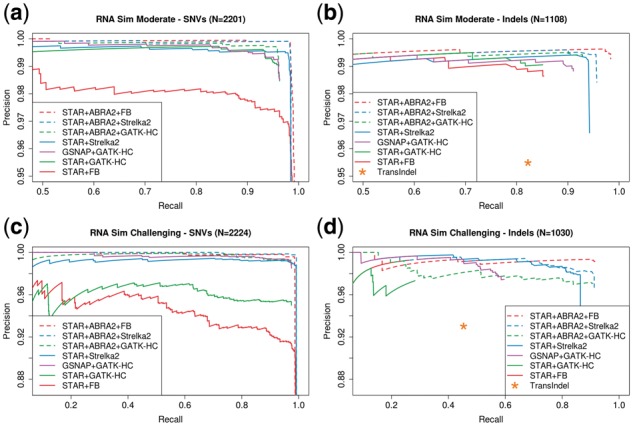
Evaluation of RNA-Seq variant calling. (**a**) Precision and recall of RNA-Seq variant calling for SNPs on a simulated dataset of moderate difficulty containing indels ranging in length from 1 to 19 bp. Freebayes, Strelka2 and GATK-HaplotypeCaller (GATK-HC) are used in this evaluation. Freebayes, Strelka2 and GATK-HC are run with and without ABRA2 on STAR alignments. GATK-HC is also run on GSNAP alignments and Transindel is run on bwa-mem alignments. ABRA2 results in improvements for Freebayes, Strelka2 and GATK-HC in RNA SNP detection. (**b**) Precision and recall of RNA-Seq variant calling for indels on the moderate difficulty RNA-Seq dataset. A clear improvement in accuracy is observed in the ABRA2 realignments for Strelka2, Freebayes and GATK-HC. (**c**) Precision and recall of RNA-Seq variant calling for SNPs on a more challenging dataset containing indels ranging in length from 1 to 75 bp. ABRA2 results in substantial improvements in SNP detection for both GATK-HC and Freebayes with improvements for Strelka2 also observed. (**d**) Precision and recall of RNA-Seq variant calling for indels on the challenging dataset. Here, the 3 ABRA2 pipelines produce the best results along with Strelka2

ABRA2’s impact on variant calling on RNA-Seq data was evaluated using two sets of reads generated using the BEERs simulation engine (Grant *et al.*, 2011). We modified BEERs to generate reads containing indels of length 1–19 bp for a dataset of moderate difficulty and 1–75 bp for a more challenging dataset. Variants were called using Freebayes, GATK-HC and Strelka2 in germline mode, both with and without ABRA2 run on STAR alignments. Transindel was run against the STAR alignments and GATK-HC was additionally run against GSNAP alignments (Wu *et al.*, 2016). On the moderate dataset, ABRA2 enables improvements for Freebayes, Strelka2 and GATK-HC in SNP detection (Fig. 3a). For indels, a clear improvement in recall is observed in the ABRA2 realignments for Freebayes, Strelka2 and GATK-HC (Fig. 3b). On the challenging dataset, ABRA2 results in substantial improvements in SNP detection for both GATK-HC and Freebayes as well as a noticeable improvement for Strelka2 (Fig. 3c). The improvement in SNP detection accuracy in many cases was due to misalignments where the longer indel lengths cause problems for the variant callers in the non-ABRA2 cases. For indels, the 3 ABRA2 configurations yield the best results along with Strelka2 (Fig. 3d).

### 3.3 Genome in a bottle assessment

**Fig. 4.**
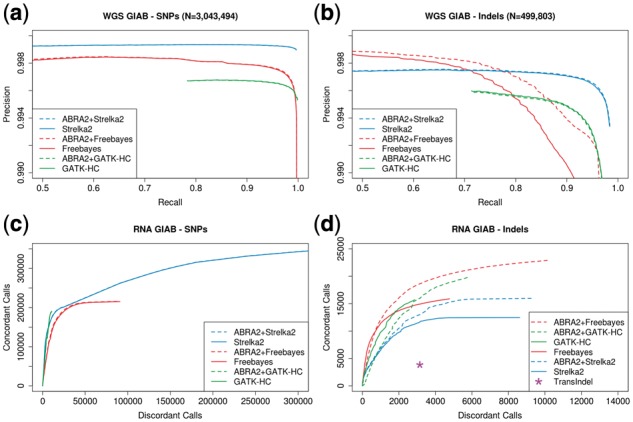
Genome in a bottle assessment. (**a**) Precision and recall of whole genome DNA germline SNP variant calling on the Genome in a Bottle dataset. Freebayes, Strelka2 and GATK-HC were run with and without ABRA2. (**b**) Precision and recall of whole genome DNA germline indel variant calling on the Genome in a Bottle dataset. ABRA2 improves accuracy for Freebayes and has a small impact on Strelka2 and GATK-HC. (**c**) Evaluation of RNA-Seq SNP variant calling on the Genome in a Bottle dataset. In the absence of ground truth for RNA-Seq, we report counts of calls concordant and discordant with the Genome in a Bottle truth set. Strelka2, Freebayes and GATK-HC were run with and without ABRA2 while TransIndel was run without ABRA2 only. (**d**) Evaluation of RNA-Seq indel variant calling on the Genome in a Bottle dataset. ABRA2 improves overall performance for both Freebayes and Strelka2 as well as recall for GATK-HC with the ABRA2+Freebayes combination achieving the highest accuracy

We next evaluated ABRA2’s performance using Genome in a Bottle (GIAB) (Zook *et al.*, 2016) data for subject NA12878. Genotypes were called using Freebayes, Strelka2 and GATK-HC both with and without ABRA2 on a whole genome dataset. ABRA2 has little impact on SNP detection across the three methods (Fig. 4a). For indels, ABRA2 enables a clear improvement in Freebayes accuracy, and has a small impact on GATK-HC and Strelka2 (Fig. 4b). We additionally acquired RNA-Seq reads for the same subject from Gene Expression Omnibus (Sample GSM2308414) (The ENCODE Project Consortium, 2012) for evaluation. Variants were called using Freebayes, Strelka2 and GATK-HC both with and without ABRA2. TransIndel was also used for variant calling without ABRA2. In the absence of ground truth specifically in the context of RNA-Seq, we report the number of called alleles that are concordant and discordant with the GIAB whole genome truth set. Among unfiltered calls, SNPs that fit the profile of A-to-I RNA editing dominate the discordant call set with C-to-U calls also being somewhat elevated. By contrast, the transition/transversion ratio for the concordant SNPs is 2.2 (Supplementary Fig. S4b, c). To address the impact of these potential RNA edits on the discordant calls, SNPs found in the Rigorously Annotated Database of A-to-I RNA Editing (RADAR) (Ramaswami and Li, 2014) were filtered which resulted in a substantial reduction of discordant calls (i.e. 79% of discordant A-to-I calls were removed for ABRA2+Freebayes at QUAL 30 and a total of 8 concordant calls were removed) (Supplementary Fig. S4a). ABRA2 has a small impact on SNP detection in this dataset (Fig. 4c), and enables a substantive improvement in overall performance in indel detection for both Freebayes and Strelka2 with an increase in concordant calls also observed in GATK-HC (Fig. 4d).

### 3.4 EGFR deletions in TCGA lung adenocarcinoma

**Fig. 5.**
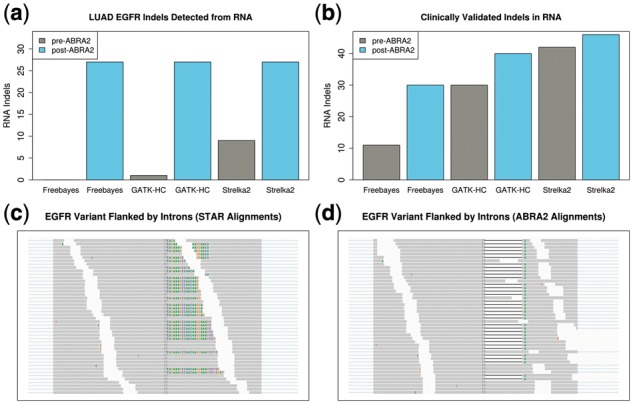
TCGA LUAD EGFR deletions and cancer gene panel clinical validation. (**a**) Number of clinically actionable TCGA EGFR exon 19 indels detected from RNA alone by GATK-HC, Freebayes and Strelka2. All callers were run both with and without ABRA2. ABRA2 enabled detection of all 27 deletions that were originally detected in DNA by each of the evaluated callers. (**b**) Number of clinically validated indels from the UNCSeq project detected from RNA alone. ABRA2 increases the number of indels detected in RNA by Freebayes, GATK-HC and Strelka2. (**c**) STAR alignments of a complex EGFR variant flanked by introns. Reads containing the variant are soft clipped and the alignments do not accurately reflect the variant. (**d**) Complex EGFR variant flanked by introns and revealed by ABRA2

Deletions in Epidermal Growth Factor Receptor (EGFR) exon 19 have oncogenic potential and can be indicators for treatment with Gefitinib or Erlotinib, making accurate detection of these variants clinically vital. Deletions ranging in length from 9 to 24 bp were detected in 23 samples of the TCGA Lung Adenocarcinoma (LUAD) cohort using matched tumor/normal DNA samples (The Cancer Genome Atlas Network, 2014). Additionally, Ye *et al.* identified 8 complex indels (Ye *et al.*, 2016) in the same cohort with 3 overlapping the TCGA set for a total of 28 cases, of which 27 have available RNA-Seq data. We attempted here to detect these deletions from RNA-Seq alone. Variant calling pipelines were run as described in the GIAB RNA-Seq experiment. GATK-HC, Freebayes and Strelka2 detect 1, 0 and 9 deletions respectively without ABRA2. Using the ABRA2 alignments, all callers detect all 27 deletions (Fig. 5a). Non-trivial variants in this exon prove to be particularly difficult to accurately identify due to nearby introns (Fig. 5c–d).

### 3.5 Clinically validated indels

RNA sequencing was performed on 67 subjects that were previously found to harbor 88 coding indels in cancer genes via DNA matched tumor/normal sequencing as part of the UNCSeq project (Jeck *et al.*, 2014; Patel *et al.*, 2018). These 88 variants were all confirmed as part of standard of care molecular testing in the Molecular Pathology and Genetics laboratory at UNC Medical Center. Variant calling pipelines on RNA-Seq data for these 67 subjects were run as previously described. ABRA2 increases the number of these clinically confirmed indels detected from RNA-Seq alone across all three variant callers (Fig. 5b).

### 3.6 TCGA breast and lung adenocarcinoma

We used ABRA2 and Cadabra to detect somatic indels in 1068 TCGA Breast (BRCA) and 506 Lung Adenocarcinoma (LUAD) subjects with matched tumor/normal DNA and tumor RNA (The Cancer Genome Atlas Network, 2012; The Cancer Genome Atlas Research Network, 2014). We additionally ran ABRA2 and Cadabra on 98 whole genome tumor/normal DNA pairs in the BRCA cohort.

**Fig. 6.**
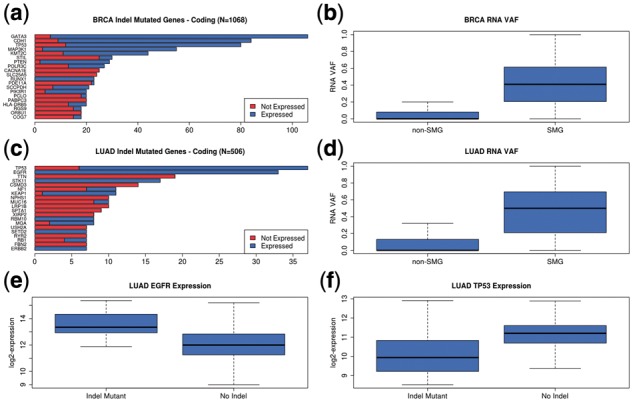
TCGA breast and lung adenocarcinoma indels. (**a**) Frequency of genes harboring somatic coding indels in the TCGA BRCA cohort with evidence of expression of the indel mutation. Frequency is computed by simple counts of subjects containing coding mutations and does not take into account exon lengths of each gene. (**b**) Comparison of RNA Variant Allele Frequency (VAF) of coding indels between genes previously identified as ‘significantly mutated’ (SMG) and not significantly mutated (non-SMG) in the BRCA cohort. (**c**) Frequency of genes harboring somatic coding indels in the TCGA LUAD cohort with evidence of expression of the indel mutation. (**d**) Comparison of RNA VAF of coding indels between genes previously identified as ‘significantly mutated’ and not significantly mutated in the LUAD cohort. (**e**) EGFR gene expression for tumor RNA samples containing an EGFR coding indel versus those without an EGFR coding indel in the TCGA LUAD cohort. EGFR is more highly expressed in samples containing an indel. (**f**) TP53 gene expression for tumor RNA samples containing a TP53 coding indel versus those without a TP53 indel in the TCGA LUAD cohort. TP53 expression is lower in samples containing an indel

954 and 473 subjects contained at least one somatic protein coding indel in the BRCA and LUAD cohorts respectively. Detected deletions ranged in size from 1 to 461 bases with a 75th percentile of 9 bases, while insertions ranged in size from 1 to 242 bases with a 75th percentile of 3 bases. In the BRCA cohort GATA3, TP53, CDH1 and MAP3K1 contained somatic indels in more than 5% of subjects (106, 84, 80 and 55 subjects respectively). Evidence of expression of the indel mutations for each of these genes is present in 85% of subjects or more (94, 89, 85, 95%) (Fig. 6a). In the LUAD cohort, somatic indels were detected in TP53 and EGFR in more than 5% of samples (37 and 33 subjects respectively). 84% of the TP53 indels were expressed and 100% of the EGFR indels were expressed (Fig. 6b). More generally, genes previously identified as significantly mutated in the TCGA studies were more likely to exhibit increased variant allele frequency (Fig. 6b, d) of indels. Notably, 100% of the LUAD EGFR indels are in frame whereas 84% of the LUAD TP53 indels are frameshift variants. Additionally, elevated gene expression levels were observed in the LUAD EGFR indel mutated samples suggesting possible oncogenic activity (Fig. 6e). By contrast, TP53 gene expression was reduced in LUAD TP53 indel mutated subjects indicating a possible disruption of tumor suppression activity (Fig. 6f).

For these TCGA datasets, ABRA2 was run using 16 cores for WXS and RNA-Seq processing. Median runtimes for WXS tumor/normal joint realignment and RNA-Seq realignment were 40 min and 5.1 h and RAM usage was roughly 16GB and 24GB respectively. WGS tumor/normal pairs were realigned using 32 cores with a median runtime of 15.5 h using 60GB of RAM or less.

## 4 Discussion

Variant detection and identification of indels are important for both research and clinical diagnoses in a variety of areas including Cancer and Mendelian disorders. The ability to accurately identify indels in DNA has been an area of focus by multiple groups and good progress has been made. Detecting variants that have been expressed in RNA-Seq enables greater insight into function. In the context of cancer diagnoses and research, detecting expressed mutations has the potential of enabling better understanding of mutations with oncogenic potential, identification of neoantigens and potential assessment of mutational burden.

ABRA2 improves upon the original ABRA implementation enabling increased accuracy of indel detection in DNA via realignment of NGS reads. ABRA2 additionally improves upon ABRA in the areas of speed and scalability. ABRA2 was designed with RNA-Seq in mind and does not require special processing to treat RNA-Seq data as if it were DNA. By directly making use of splice junction information, ABRA2 is able to achieve greatly improved accuracy over other methods. Alignment of each contig to each putative transcript is currently the computational bottleneck for ABRA2 and we believe this coud potentially be optimized by using a graph representation (Garrison *et al.*, 2018; Paten *et al.*, 2017) for contig alignment instead of distinct linear representations of each transcript.

ABRA2 can also be used to realign individual samples or multiple samples jointly such as in the case of matched tumor/normal pairs. Indels identified in DNA can optionally be used to inform RNA alignments although RNA can also be processed independently. As we have shown in several assessments on both real and simulated data, ABRA2 can be used to improve alignments in a variety of scenarios including both germline and somatic variant calling as well as targeted sequencing, whole genomes and transcriptomes. The improved alignments produced by ABRA2 enhance variant detection by a variety of downstream tools with substantial improvement demonstrated for indel detection in both DNA and RNA. This method should lead to the identification of additional patients with previously undetected somatic mutations and indels as well as improving assessment of expressed variants, thus leading to improvements in patient care and precision medicine.

## 5 Methods

### 5.1 Exome simulation

To generate DNA exome simulated data, trimmed fastq files for subject NA12878 were downloaded from: ftp://ftp-trace.ncbi.nih.gov/giab/ftp/data/NA12878/Garvan_NA12878_HG001_HiSeq_Exome. Bamsurgeon (Ewing *et al.*, 2015) was used to insert SNVs and indels into exome targeted reads. The output of Bamsurgeon is a normal BAM without the simulated variants and a tumor BAM containing the simulated variants. Inserted sequences include repeats, mobile elements and randomly generated sequence. Indel sizes range from 1 to 100 bp.

### 5.2 RNA-Seq simulation

Simulated RNA-Seq data were generated using the BEERs simulation engine. We modified BEERs to generate reads containing indels of length 1–19 bp for a dataset of moderate difficulty and 1–75 bp for a more challenging dataset. For the challenging dataset, the STAR aligner was configured to use a minimum intron length of 76.

### 5.3 Read prep and alignment

Initial DNA alignments were performed using bwa-mem version 0.7.9a and 0.7.16a. RNA-Seq alignments were performed using STAR version 2.5.3 in two pass mode with unmapped reads included in the output and Gencode annotations provided to the aligner. Parameters outFilterScoreMinOverLread and outFilterMatchNminOverLread were set to .45, which allows STAR to align only one end of a read pair when alignment for both ends is not possible. STAR output was post-processed to assign unmapped reads to the locus of its mapped mate when applicable as is the recommended practice per the Sequence Alignment/Map Format Specification (https://samtools.github.io/hts-specs/SAMv1.pdf). For the challenging RNA simulation, alignIntronMin is set to 76. When applicable, STAR was run separately with outSAMmapqUnique set to 60 which was required to allow processing by the GATK. GSNAP version 2017-11-15 is additionally used for comparison purposes in the RNA simulations. Prior to alignment, read trimming for TCGA DNA reads was performed using SeqPurge version 0.1-874-g426ed18 (Sturm *et al.*, 2016). Duplicates were marked using either biobambam (Tischler and Leonard, 2014) or Picard Tools.

### 5.4 Realignment with ABRA2

Realignments were performed using ABRA2 version 2.14 for most cases, with versions 2.11 and 2.12 also used for TCGA whole genome processing. In all cases the –undup flag was set, allowing reads erroneously marked as duplicates to be rescued in cases where one end of a read pair is initially mapped and both ends are mapped post-ABRA2. This requires that duplicate marking be re-run post ABRA2. For somatic cases, the tumor and normal are realigned together. For whole genomes, regions of abnormally high depth with a read count greater than 2000 were skipped. Gencode annotations and splice junctions identified in the original alignments were used to inform ABRA2 of potential junctions during RNA-Seq realignment. The maximum distance to move reads was increased to 5 00 000 and unmapped reads were not utilized in assembly. Reads containing indels abutting splice junctions were filtered as necessary to allow downstream variant callers to run to completion. For the TCGA analysis, somatic indels identified in DNA were used to inform the RNA-Seq ABRA2 realignments. Known indels were not utilized in any of the other analyses with the exception of the known indel assessment on the exome simulation.

### 5.5 Variant calling

Evaluated variant caller versions are Strelka2 (version 2.9.2), Freebayes (commit c15b070639d54d112988946a6902d945357e40f0), GATK HaplotypeCaller and Mutect2 (version 4.0.3.0), Lancet (version 1.0.1), Manta (version 1.2.2) and TransIndel (version 0.1). For RNA-Seq variant calling with GATK Haplotyper, the GATK version at git commit 8103bde7ef90c22e66f9f639809cc91122928ffd was used. In general, default settings are used with a few exceptions. On the exome simulations, Strelka2 maxIndelSize was set to 100. Short Tandem Repeat penalties were disabled for Cadabra on the Dream Challenge dataset. Lancet calls filtered by only the StrandBias filter are converted to PASS which appears to produce improved results on the simulated datasets as was also observed in Narzisi *et al.* For the GIAB RNA-Seq analyses, SNPs found in the RADAR database were filtered. RADAR version 2 was used and ‘lifted over’ from hg19 to hg38 (Kent *et al.*, 2002). For the TCGA analysis, DNA indels were called using Cadabra and RNA indel calling was performed independently using a beta binomial test based upon the UNCeqR implementation (Wilkerson *et al.*, 2014). Variants arising from low complexity regions were filtered as described by Li (2014). Variant calls were annotated using VEP (McLaren *et al.*, 2016). For each variant caller, a single metric was used for thresholding values in the precision/recall plots. For Mutect2 the TLOD score was used. For somatic Strelka2 the QSS_NT and QSI_NT scores were used for SNVs and indels respectively. For Cadabra, Lancet, Freebayes, GATK-HC and germline Strelka2 the QUAL score was used. TransIndel did not provide a statistic indicating variant call quality and we used a single point to reflect its performance.

### 5.6 Variant calling evaluation

Variant calls were evaluated using happy (https://github.com/Illumina/hap.py) combined with RTG Tools (https://github.com/RealTimeGenomics/rtg-tools) for the GIAB datasets. The default behavior of hap.py is to assess genotype accuracy. For the GIAB RNA-Seq dataset, we instead use the allele match method described by Krusche *et al.* (2018). All other datasets were evaluated using RTG Tools.

## Supplementary Material

btz033_Supplementary_Materials
