# Supplementary material for: Improved indel detection in DNA and RNA via realignment with ABRA2
Source: Bioinformatics. 2019 Jan 15;35(17):2966–73. doi: 10.1093/bioinformatics/btz033 (PMC6735753; doi:10.1093/bioinformatics/btz033)
Supplement: btz033_Supplementary_Materials [file btz033_supplementary_materials.pdf]

Supplementary Material for: Improved Indel Detection in DNA and RNA via Realignment with ABRA2

Supplemental Figure 1 – Comparison of ABRA and ABRA2

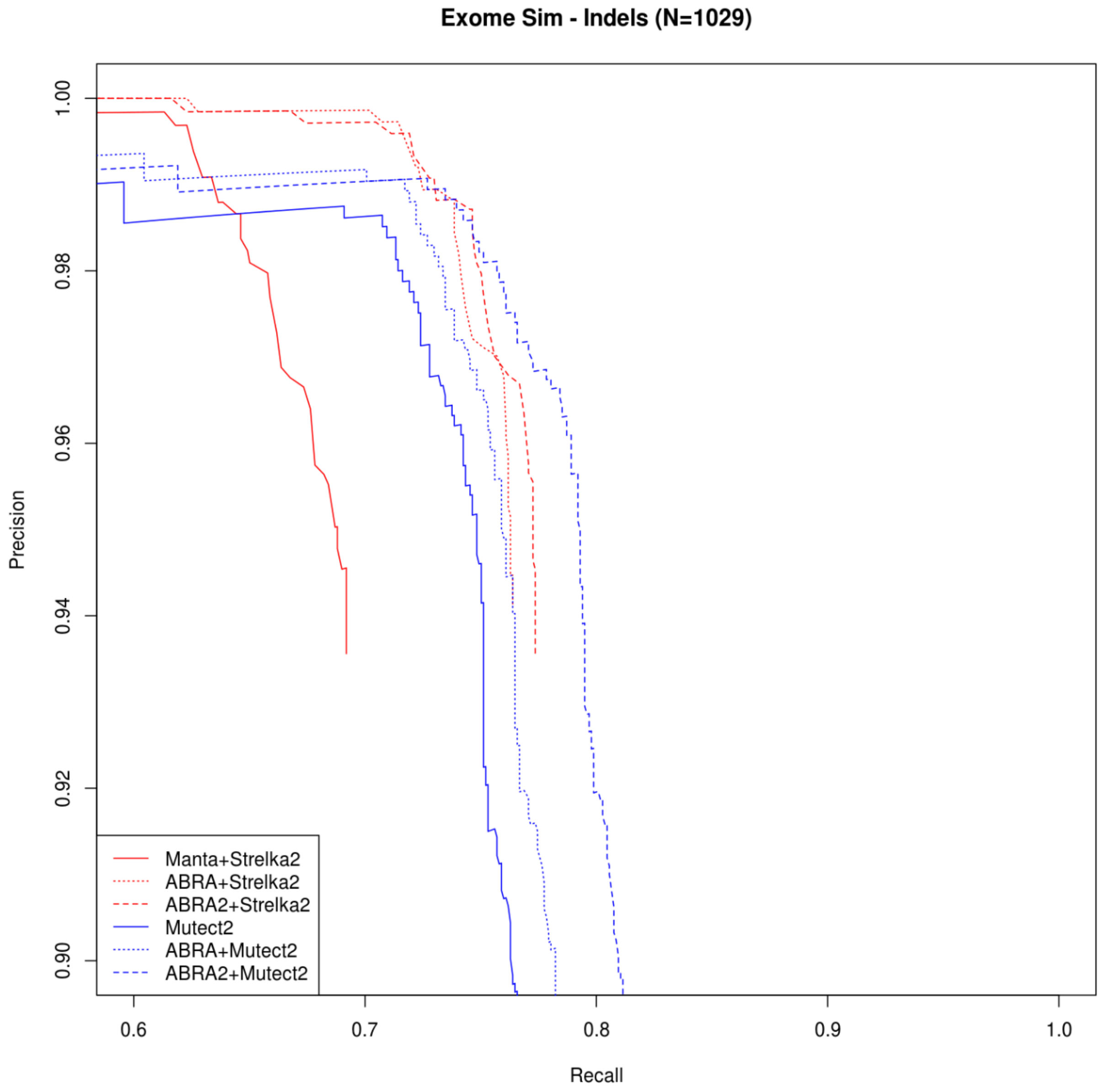

Supplemental Figure 1. Performance comparison of ABRA2 versus the original ABRA for indels on the simulated exome data.

Supplemental Figure 2 – Impact of Localized Assembly on Variant Detection

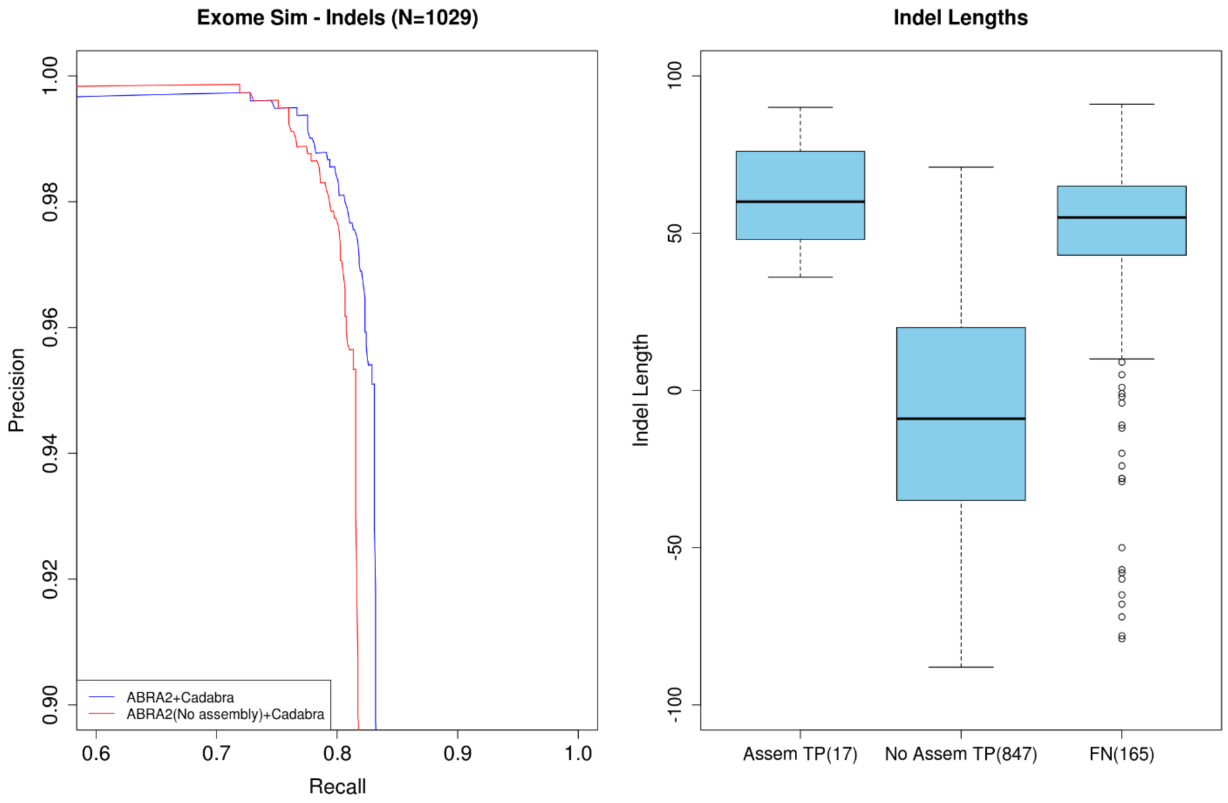

Supplemental Figure 2. Assessment of the impact of localized assembly on realignment. Localized assembly enabled detection of an additional 17 insertions with a median length of 60 bases compared to ABRA2 run with localized assembly disabled.

Supplemental Figure 3 – Impact of Known Indel Usage on Variant Detection

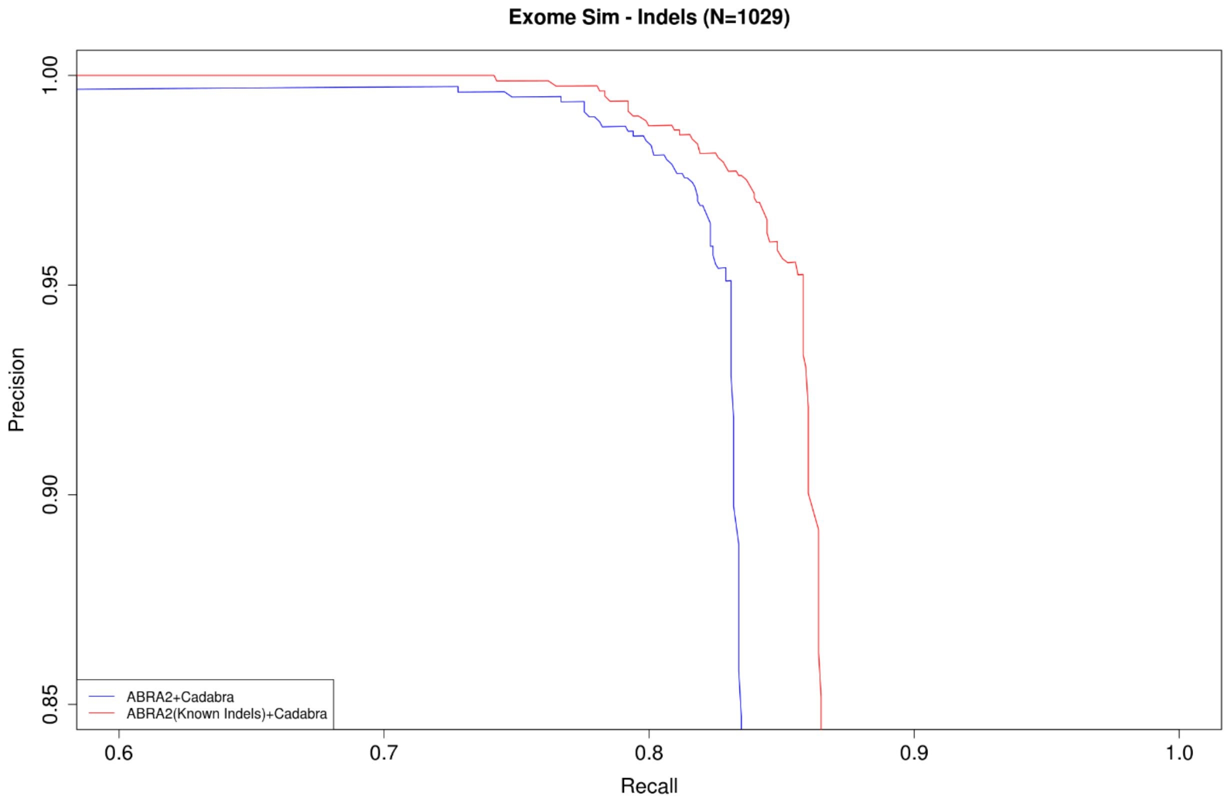

Supplemental Figure 3. Usage of known indels to inform ABRA2 realignment resulted in an additional 31 insertions detected at nucleotide resolution with a median length of 49 bases. The variant caller identified an additional 52 insertions not at nucleotide resolution with a median length 61.5 bases. The non-nucleotide resolved calls were filtered and are not included in the figure result.

Supplemental Figure 4 – Impact of RNA Editing on GIAB Variant Calling

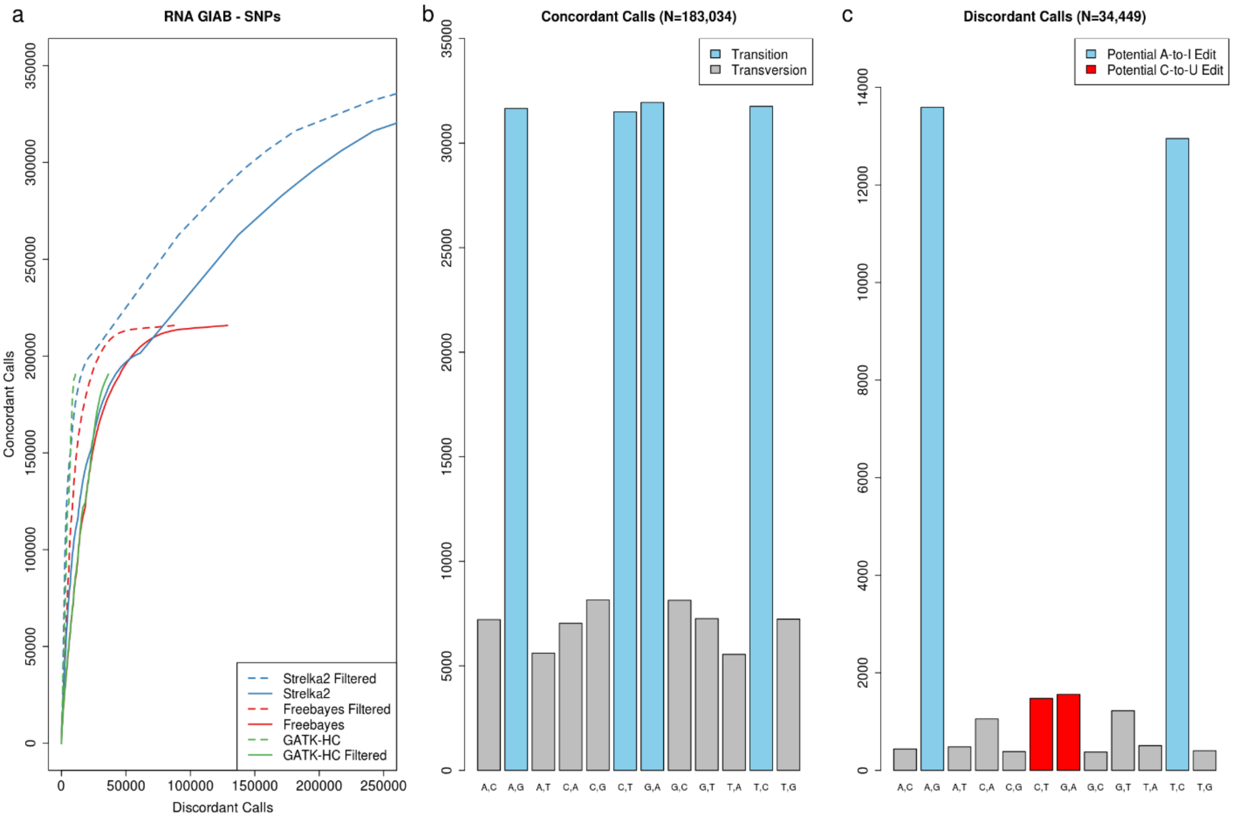

Supplemental Figure 4. RNA editing impacts the assessment of RNA SNP variant calling on the Genome in a Bottle (GIAB) dataset. (a) ROC-like plot showing concordant and discordant calls for each variant caller before and after filtering against entries in the RADAR database. All callers were run against STAR alignments realigned with ABRA2. (b) Counts of calls for each of the 12 possible SNP variants among the unfiltered concordant calls. (c) Counts of calls for each of the 12 possible SNP variants among the unfiltered discordant calls.
